# Cefazolin versus alternative antibiotics for perioperative prophylaxis in patients labeled as cephalosporin allergic

**DOI:** 10.1017/ash.2025.10221

**Published:** 2025-12-02

**Authors:** Jenna Jackson, Cristina Corsini Campioli, Richard Crockett, Erica Loomis, Kristin Cole, Diana J. Schreier, Kellie Arensman Hannan

**Affiliations:** 1 Department of Pharmacy, Children’s Minnesota, Minneapolis, MN, USA; 2 Division of Infectious Diseases, Mayo Clinic Health System, Mankato, MN, USA; 3 Division of Allery and Immunology, Mayo Clinic Health System, Mankato, MN, USA; 4 Department of Surgery, Mayo Clinic, Rochester, MN, USA; 5 Department of Quantitative Health Sciences, Mayo Clinic, Rochester, MN, USA; 6 Department of Pharmacy, Mayo Clinic, Rochester, MN, USA

## Introduction

Cefazolin is a preferred antibiotic for perioperative prophylaxis due to its spectrum of activity, efficacy, tolerability, affordability, and ease of administration.^1^ The use of alternative agents such as vancomycin or clindamycin is associated with higher risk of surgical site infections (SSIs), adverse effects, and higher cost.^2^ Previous studies have demonstrated that cefazolin can be safely used in patients with penicillin allergy labels; however, data on the safety of cefazolin use in patients with cephalosporin allergy labels remains limited.^3,4^ The purpose of this study is to investigate the incidence of immediate hypersensitivity reactions (HSRs) and SSIs with cefazolin compared to vancomycin or clindamycin in patients with cephalosporin allergy labels when used for perioperative prophylaxis.

## Methods

This is a retrospective analysis of adults with a non-cefazolin cephalosporin allergy label who underwent a surgical procedure at Mayo Clinic between July 2022 and September 2022 and received perioperative prophylaxis with cefazolin, clindamycin, or vancomycin. Incidence of immediate HSRs and postoperative infections were determined using manual chart review. Patients were excluded if they were pregnant, incarcerated, received more than one perioperative antibiotic, received antibiotics for non-perioperative indications within 24 hours of the procedure, had a documented allergy label to cefazolin or ceftezole, or declined research authorization. The Mayo Clinic Institutional Review Board deemed this study to be exempt.

Cephalosporin allergy labels were classified as high risk (including Stevens-Johnson syndrome, toxic epidermal necrolysis, or multiorgan hypersensitivity response), moderate risk (including disseminated hypersensitivity or anaphylaxis), or low risk (including side effects/intolerances [gastrointestinal intolerance, headache, cough, hypertension, tinnitus, muscle aches, or neuropsychiatric disturbances], limited HSRs [self-limited cutaneous rash or itching], or nonspecific).^5^ For preexisting cephalosporin allergy labels, type-1 HSRs were defined as angioedema, edema, breathing difficulties, wheezing, urticaria, hives, or hypotension. For the primary outcome, immediate perioperative HSRs were classified as probable or possible. Probable immediate HSR we defined as the presence of acute hypotension, angioedema, bronchospasm, erythema, flushing, increased mechanical ventilator pressures, respiratory distress, urticaria, wheezing, or allergic reaction noted.^6^ Possible immediate HSR was determined by the administration of epinephrine and/or diphenhydramine, with doses and routes consistent with HSR treatment, on the day of surgery.^6^ Consideration for immediate HSR began when perioperative antibiotics were administered and continued for 60 minutes following the conclusion of surgery. SSIs were classified as superficial, deep, and organ space according to National Healthcare Safety Network surveillance requirements.^7^

Categorical data was summarized using total counts and percentages, and continuous data was summarized using means and standard deviations. To compare baseline characteristics and outcomes between groups, χ^2^ or Fisher’s exact tests were for categorical data, and Analysis of Variance for continuous data. All analyses were performed using SAS version 9.4 software (SAS Institute, Inc.; Cary, NC), and p-values ≤ .05 were considered statistically significant.

## Results

A total of 406 surgical cases were included. Patient baseline characteristics were similar across the three groups, with a notable difference being the selection of perioperative antibiotic based on patient allergy type (Supplementary Table 1). Most patients who received cefazolin had allergy types that were classified as low risk, whereas patients with allergies classified as moderate or high risk were more likely to receive vancomycin or clindamycin.

No probable immediate HSR occurred. Possible immediate HSRs occurred in three (1.7%) cases where cefazolin was used, six (6.5%) cases with clindamycin, and seven (5%) cases with vancomycin perioperative prophylaxis (*p*-value = .12) (Table 1). There was one documented SSI, which occurred in a patient who had received vancomycin perioperatively.

**Table 1.** Outcomes

		Total, *n* = 406	CZO, *n* = 173	CLI, *n* = 93	VAN, *n* = 140	*P* value
Immediate HSR	Probable	0 (0%)	0 (0%)	0 (0%)	0 (0%)	0.12
	Possible	16 (3.9%)	3 (1.7%)	6 (6.5%)	7 (5%)	
	None documented	390 (96.1%)	170 (98.3%)	87 (93.5%)	133 (95%)	
Possible immediate HSR	Total	16 (3.9%)	3 (1.7%)	6 (6.5%)	7 (5%)	0.12
	DPH	8 (50%)	2 (67%)	0 (0%)	6 (86%)	0.008
	Epinephrine	8 (50%)	1 (33%)	6 (100%)	1 (14%)	
Corticosteroid administration within 24 hours of surgery		291 (71.7%)	118 (68.2%)	69 (74.2%)	104 (74.3%)	0.41
Postoperative SSI—superficial		1 (0.2%)	0 (0%)	0 (0%)	1 (0.7%)	0.57
Subgroup with history of cephalosporin type-1 HSR		Total, *n* = 152	CZO, *n* = 27	CLI, *n* = 45	VAN, *n* = 80	P value
Immediate HSR	Probable	0 (0%)	0 (0%)	0 (0%)	0 (0%)	0.29
	Possible	8 (5.3%)	0 (0%)	4 (9%)	4 (5%)	
	None documented	144 (94.7%)	27 (100%)	41 (91%)	76 (95%)	

CZO, cefazolin; CLI, clindamycin; DPH, diphenhydramine; HSR, hypersensitivity reaction; SSI, surgical site infection; VAN, vancomycin.

The primary outcome was also assessed in a subgroup of 152 surgical cases for patients with a history of type-1 hypersensitivity to a cephalosporin. No possible or probable immediate HSRs occurred when cefazolin was used; however, 4 (9%) of cases where clindamycin was used and 4 (5%) of cases where vancomycin was used had a possible immediate HSR (*P* = .29).

## Discussion

Among patients with cephalosporin allergy labels, the use of perioperative cefazolin was not associated with an increased risk of immediate HSR compared to clindamycin or vancomycin. This finding is consistent with previous studies of patients with penicillin allergy labels.^2,3^ It is logical given that cefazolin does not share an R-group side chain with any other beta-lactam antibiotics available in the United States.^8,9^

Our cohort includes 173 surgical cases where cefazolin was given to patients with cephalosporin allergy labels, with an observed rate of possible immediate HSR lower than that for cases where clindamycin or vancomycin were used. Among these, cefazolin was used for 27 surgical cases for patients with a history of type-1 HSR to a cephalosporin, and no immediate HSRs were observed. The higher incidence of immediate HSR among cases where clindamycin or vancomycin was used, as compared to cefazolin, may be due to individuals with drug allergies being predisposed to having additional drug allergies regardless of any structural similarity between the drugs.^10^

Limitations of this study include its retrospective design and modest sample size. Our assessment of HSRs relied on documentation within the electronic medical record. HSRs observed may have been caused by perioperative medications other than the antibiotic. Use of corticosteroids could have muted potential HSRs to an antibiotic; however, corticosteroid administration was not different between groups.

Our results suggest that avoiding cefazolin for perioperative prophylaxis in patients with non-cefazolin cephalosporin allergy labels may not be warranted.

## Supporting information

10.1017/ash.2025.10221.sm001 Jackson et al. supplementary material Jackson et al. supplementary material

## Supplementary material

The supplementary material for this article can be found at https://doi.org/10.1017/ash.2025.10221.
