# Supplementary material for: Cefazolin versus alternative antibiotics for perioperative prophylaxis in patients labeled as cephalosporin allergic
Source: Antimicrob Steward Healthc Epidemiol. 2025 Dec 2;5(1):e326. doi: 10.1017/ash.2025.10221 (PMC12722541; doi:10.1017/ash.2025.10221)
Supplement: Jackson et al. supplementary material [file S2732494X25102210sup001.docx]

Supplementary Table 1. Population baseline characteristics

|  | | Total, n=406 | CZO,  n=173 | CLI,  n=93 | VAN, n=140 | P value |
| --- | --- | --- | --- | --- | --- | --- |
| Sex | Male | 110 (27.1%) | 51 (29.5%) | 19 (20.4%) | 40 (28.6%) | 0.32 |
|  | Female | 295 (72.7%) | 122 (70.5%) | 74 (79.6%) | 99 (70.7%) |  |
|  | Other | 1 (0.2%) | 0 (0%) | 0 (0%) | 1 (0.7%) |  |
| Age, years, Mean (SD) | | 60.6 (16.4) | 63.2 (17.1) | 55.4 (17.2) | 60.8 (14.0) | <0.001 |
| Race | White | 391 (96.3%) | 170 (98.3%) | 87 (93.5%) | 134 (95.7%) | 0.27 |
|  | Black | 7 (1.7%) | 1 (0.6%) | 4 (4.3%) | 2 (1.4%) |  |
|  | Asian | 5 (1.2%) | 1 (0.6%) | 1 (1.1%) | 3 (2.1%) |  |
|  | Other/Unlisted | 3 (0.7%) | 1 (0.6%) | 1 (1.1%) | 1 (0.7%) |  |
| Height, cm, Mean (SD) | | 167.5 (10.1) | 166.9 (10.7) | 168.4 (9.5) | 167.5 (9.6) | 0.39 |
| Weight, kg, Mean (SD) | | 86.1 (23.6) | 86.3 (25.7) | 85.1 (21.0) | 86.6 (22.8) | 0.85 |
| Cephalosporin Allergy(s) | Cefadroxil | 25 (6.2%) | 13 (7.5%) | 4 (4.3%) | 8 (5.7%) | 0.56 |
|  | Cephalexin | 217 (53.4%) | 85 (49.1%) | 55 (59.1%) | 77 (55%) | 0.27 |
|  | Cefaclor | 60 (14.8%) | 14 (8.1%) | 16 (17.2%) | 30 (21.4%) | 0.003 |
|  | Cefoxitin | 1 (0.2%) | 0 (0%) | 1 (1.1%) | 0 (0%) | 0.19 |
|  | Cefprozil | 21 (5.2%) | 14 (8.1%) | 2 (2.2%) | 5 (3.6%) | 0.065 |
|  | Cefuroxime | 35 (8.6%) | 18 (10.4%) | 9 (9.7%) | 8 (5.7%) | 0.31 |
|  | Cefdinir | 33 (8.1%) | 18 (10.4%) | 3 (3.2%) | 12 (8.6%) | 0.12 |
|  | Cefpodoxime | 6 (1.5%) | 1 (0.6%) | 2 (2.2%) | 3 (2.1%) | 0.43 |
|  | Ceftriaxone | 30 (7.4%) | 10 (5.8%) | 8 (8.6%) | 12 (8.6%) | 0.57 |
|  | Cefepime | 19 (4.7%) | 10 (5.8%) | 2 (2.2%) | 7 (5%) | 0.40 |
|  | Other Cephalosporins | 20 (8.2%) | 3 (3%) | 4 (6.9%) | 13 (15.1%) | 0.010 |
| Allergy Type | Low | 253 (62.3%) | 146 (84.4%) | 48 (51.6%) | 59 (42.1%) | <0.001 |
|  | Moderate | 152 (37.4%) | 27 (15.6%) | 45 (48.4%) | 80 (57.1%) |  |
|  | High | 1 (0.2%) | 0 (0%) | 0 (0%) | 1 (0.7%) |  |
| Allergic Reaction | Anaphylaxis | 22 (5.4%) | 3 (1.7%) | 6 (6.5%) | 13 (9.3%) | 0.012 |
|  | Breathing difficulties | 37 (9.1%) | 7 (4%) | 7 (7.5%) | 23 (16.4%) | <0.001 |
|  | GI intolerance | 75 (18.5%) | 57 (32.9%) | 7 (7.5%) | 11 (7.9%) | <0.001 |
|  | Hives | 96 (23.6%) | 15 (8.7%) | 33 (35.5%) | 48 (34.3%) | <0.001 |
|  | Rash | 168 (41.4%) | 63 (36.4%) | 47 (50.5%) | 58 (41.4%) | 0.083 |
|  | Itching | 44 (10.8%) | 19 (11%) | 9 (9.7%) | 16 (11.4%) | 0.91 |
|  | Edema/Swelling | 20 (4.9%) | 6 (3.5%) | 5 (5.4%) | 9 (6.4%) | 0.47 |
|  | Other | 53 (13.1%) | 23 (13.3%) | 10 (10.8%) | 20 (14.3%) | 0.73 |
|  | Unknown or undocumented | 17 (4.2%) | 12 (6.9%) | 3 (3.2%) | 2 (1.4%) | 0.047 |

Abbreviations: CZO, cefazolin; CLI, clindamycin; GI, gastrointestinal; SD, standard deviation; VAN, vancomycin (VAN).
